# W. G. A. Bonwill

**Published:** 1899-11

**Authors:** Rodrigues Ottolengui


### Obituary.

W. G. A. BONWILL DEAD.—Dr. W. G. A. Bonwill is dead. In him the profession loses one of its most illustrious men. I lose one of my warmest and most admired friends. It was an advantage to know him and a privilege to be received by him in his home, at all times as I was, as an equal, irrespective of age and personal achievements.

Born in Camden, Del., Oct. 4, 1838, he died in Philadelphia, Sept. 25, 1899. Throughout all the sixty-one years of his life he faithfully lived up to the motto which he wore engraved on his ring:

*In Veritate Est Victoria.*

And yet in spite of a really affectionate disposition, and a generous willingness at all times to share with his fellows the results of the exercise of his mechanical genius towards the elevation of dentistry, he made enemies, and it was ever pitiful to hear him make such remarks as: "I am proud of the manner in which my native city has disowned me." Yet, though the statement that he made enemies is true, he likewise had innumerable staunch admirers and many real friendships. Among his papers will surely be found a sufficient number of letters of commendation from prominent men throughout the world to have satisfied even Bonwill. It may please the writers of these to know that their written words cheered his declining years and that it was a pleasure to him to read aloud these letters to friends such as I, who might spend a night with him and be willing to sit up into the small hours. And now he has passed to that realm where naught but love resides, and enemies cannot reach him. *Requiescat in pace.*—RODRIGUES OTTOLENGUI, in *Items of Interest.*

